# Periorbital Facial Necrotizing Fasciitis in Adults: A Rare Severe Disease with Complex Diagnosis and Surgical Treatment—A New Case Report and Systematic Review

**DOI:** 10.3390/jpm13111612

**Published:** 2023-11-16

**Authors:** Mihaela Pertea, Madalina-Cristina Fotea, Stefana Luca, Dan Cristian Moraru, Alexandru Filip, Doinita Olinici-Temelie, Sorinel Lunca, Adrian Claudiu Carp, Oxana-Madalina Grosu, Alexandru Amarandei, Bogdan Veliceasa

**Affiliations:** 1 Faculty of Medicine, “Grigore T Popa” University of Medicine and Pharmacy, 700115 Iasi, Romania; mihaela.pertea@umfiasi.ro (M.P.); stefana.luca@d.umfiasi.ro (S.L.); cristian-dan.moraru@umfiasi.ro (D.C.M.); doinita.p.olinici@umfiasi.ro (D.O.-T.); sorinel.lunca@umfiasi.ro (S.L.); adrian-claudiu.carp@umfiasi.ro (A.C.C.); oxana-madalina.grosu@umfiasi.ro (O.-M.G.); bogdan.veliceasa@umfiasi.ro (B.V.); 2 Department of Plastic Surgery and Reconstructive Microsurgery, “Sf. Spiridon” Emergency County Hospital, 700111 Iasi, Romania; alexamarandei@yahoo.com; 3 Department of Orthopaedics and Traumatology, “Sf. Spiridon” Emergency County Hospital, 700111 Iasi, Romania; 4 Department of Dermatology, “Sf. Spiridon” Emergency County Hospital, 700111 Iasi, Romania; 5 Second Surgery Clinic, Regional Institute of Oncology, 700483 Iasi, Romania

**Keywords:** periorbital necrotizing fasciitis, enucleation, fascial flap, necrosis, skin graft

## Abstract

(1) Background: Necrotizing fasciitis (NF) is a severe and aggressive pathology with a rapid progression and high mortality risk. Periocular NF is a rare condition associated with a lower mortality risk but significantly higher disabling sequelae. (2) Methods: We present the case of a 67-year-old homeless patient, a victim of assault, with multiple untreated comorbidities (diabetes mellitus, cardiac conditions, and schizophrenia) and a delayed diagnosis of periocular necrotizing fasciitis. The condition showed a cyclical evolution influenced by the existing comorbidities, and the patient underwent both surgical and medical treatment with a multidisciplinary team. Additionally, we report a systematic review of cases from the literature. (3) Results: The patient’s survival outcomes were favorable; however, the sequelae were disabling, not only concerning aesthetic aspects but also due to the loss of the affected eye globe. The systematic review revealed the rarity of such cases and the peculiarities of the presented case compared to those reported in the literature up to this point. (4) Conclusions: Understanding the signs, symptoms, and predisposing factors, as well as the potential rare localizations of NF, including the periocular region, can lead to the early diagnosis and treatment with good functional and aesthetic outcomes, minimizing significant disabilities.

## 1. Introduction

Necrotizing fasciitis (NF) is a rare, progressive, and potentially life-threatening infection affecting the soft tissues. It is characterized by extensive necrosis of the fascial planes and surrounding tissues [1,2,3]. While the first specific description of NF can be attributed to Hippocrates, it was not until 1871 that Jones provided the initial modern account of the condition. Subsequently, in 1952, Wilson introduced the term “necrotizing fasciitis” [2,3,4].

The overall incidence of NF is estimated to be approximately 3.5 cases per 100,000 individuals [3]. The mortality rate associated with NF ranges from 10% to 40%, but, without prompt medical or surgical intervention, it can rise to as high as 80% [3,4,5,6]. While NF typically affects the extremities, abdominal wall, and perineum, cervico-facial involvement is exceptionally rare [1,7,8].

Similar to other infections, NF involves the interplay of a precipitating event, an infectious agent, and the host. Among the precipitating events, odontogenic infections are the most commonly associated cause, and Group A Streptococcus is the predominant infectious agent [3]. The pathogenesis of NF is characterized by the invasion of bacteria into the subcutaneous tissues, rapid horizontal spread of infection along the deep fascial planes, and release of bacterial toxins, leading to tissue ischemia and liquefactive necrosis [5,6,9,10].

Diagnosing NF at an early stage can be challenging due to the absence of specific symptoms, often resulting in missed diagnoses by healthcare practitioners. Left untreated, NF can rapidly progress to septic shock and result in fatalities. Therefore, upon suspicion of NF, immediate management should include resuscitation tailored to the patient’s individual needs, administration of broad-spectrum intravenous antibiotics, and early surgical intervention [6,11,12,13,14].

We present the case of an elderly, polymorbid, homeless patient who, following a traumatic incident (assault), presented with signs and symptoms indicative of oculo-palpebral fasciitis, with the diagnosis confirmed through imaging examination (RMI). The patient underwent surgical intervention in multiple stages. Given the rarity of this condition, which is seldom encountered and documented in the medical literature, we have included this case in a systematic review that comprehensively analyzes the demographic characteristics, epidemiological trends, etiological factors, mechanisms of lesion development, associated systemic disorders, initial clinical presentations, diagnostic modalities, and treatment approaches for cases of facial necrotizing fasciitis reported over the past decade. Our objective is to advance our understanding of this intricate and severe condition while furnishing valuable insights for the benefit of clinical practice.

## 2. Materials and Methods

### 2.1. Case Report

We report the case of a 67-year-old homeless male patient admitted to the Plastic Surgery and Reconstructive Microsurgery Department of “St. Spiridon” Emergency Hospital in 2017. Informed consent was obtained, and an information sheet on the use of the data was delivered to the patient. Clinical, biological, imagistic, and surgical data were extracted from the patient’s medical records. For publication, approval was obtained from the Hospital Ethics Committee.

According to the patient’s medical history, it was documented that he had encountered a traumatic event due to an aggressive act roughly 14 days prior to admission. This incident resulted in the formation of wounds in the zygomatic region, in left upper brow area, with extension to the scalp and left parietal region. Furthermore, the patient’s medical records revealed a history of diabetes, alcohol misuse, and intermittent treatment for psychiatric disorders, specifically schizophrenia. Initially, the patient was admitted to the Ear, Nose, and Throat (ENT) department due to anterior epistaxis.

**Figure 1:**
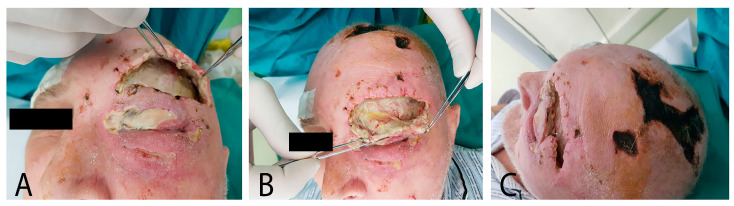
67-year-old male, with periorbital necrotizing fasciitis: (**A**) left hemifacialedema; (**B**) abundant yellowish secretions; and (**C**) area of necrosis (10/7 cm).

The patient’s overall health was significantly compromised, manifesting as pronounced edema affecting the left hemiface, particularly the upper eyebrow and eyelid, in the infraorbital regions, with extension to the occipital region, accompanied by areas of tissue necrosis. Deep-seated devitalized and infected tissues were observed in the wounds located at the left eye and upper eyelid, along with the presence of viscous, yellowish discharge. Additionally, substantial subcutaneous emphysema was noted. Furthermore, an approximately 10/7 cm area of necrosis was estimated in the vertex region of the patient’s scalp (Figure 1). Subsequently, the patient was transferred to the Plastic and Reconstructive Microsurgery Clinic.

Laboratory examinations revealed the presence of anemia and thrombocytosis.

**Figure 2:**
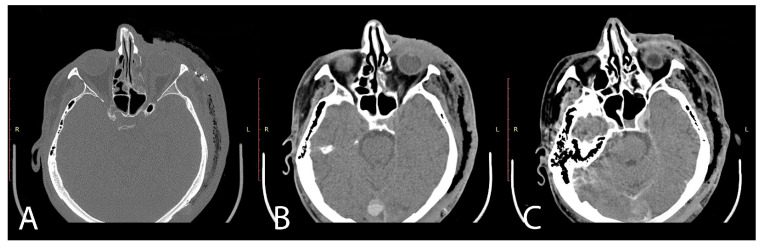
MRI examination: (**A**–**C**) subcutaneous emphysema and edema of the left hemiface.

Emergency imaging, conducted via magnetic resonance imaging (MRI) upon the patient’s admission to the clinic, confirmed the presence of subcutaneous emphysema and significant edema in the left hemiface. Remarkable symmetry in terms of fascial thickness and characteristics was observed, alongside significant alterations in the subcutaneous cellular tissue (Figure 2).

**Figure 3:**
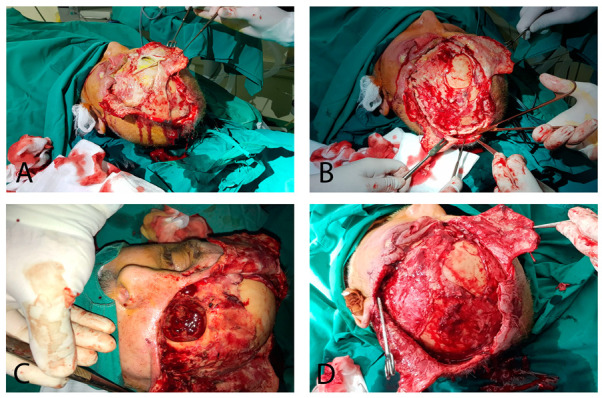
Surgical approach: (**A**) necrectomy; (**B**) extensive fasciotomy; (**C**) enucleation of the left eyeball; and (**D**) bone exposure (result after extensive debridement).

Given the patient’s local clinical presentation and overall medical condition, an urgent surgical intervention was necessary (CK = 2150/U/L, CRP = 43.11/mg/dL, WBC = 20.57 × 10^3^/µL). Under general anesthesia with orotracheal intubation, a multidisciplinary surgical approach involving plastic surgeons and ophthalmologists was executed. The surgical procedure comprised several pivotal stages: in the initial phase, extensive surgical debridement was performed, involving the removal of all necrotic and devitalized tissues, including the enucleation of the left eyeball, fasciectomy, and parieto-occipital necrectomy. Subsequently, this intervention exposed the parietal bone (Figure 3). The excised pieces were sent for histopathological examination, confirming the diagnosis.

The Laboratory Risk Indicator for Necrotizing Fasciitis (LRINEC) scoring system was employed for diagnostic evaluation, yielding a score of 9, indicative of a high-risk condition. Additionally, wound secretions were collected for microbiological examination to identify the causative pathogen and perform an antibiotic sensitivity test in preparation for targeted antibiotic therapy.

Following the fifth day of postoperative care, during which the patient’s overall health had significantly improved and achieved a state of balance, a secondary suture was performed. Subsequently, we proceeded with the coverage of the exposed parietal bone using a rotation fascial flap and a free split-thickness graft, using a similar approach as in orbital cases.

The patient remained stabilized throughout this period and received treatment for all diagnosed conditions. However, due to the patient’s unstable psychiatric status, despite the treatment administered in the hospital, he exhibited psychomotor agitation and engaged in self-mutilation for a brief but consequential period. This self-harming behavior resulted in injuries to the temporal fascial flap, necessitating subsequent surgical interventions.

The postoperative outcome following the initial surgical intervention demonstrated favorable results, with effective coverage of the defects in the osseous partimosis. A satisfactory progression was observed after eradicating the infectious focus and the coverage of the exposed bone with a fascial flap, subsequently covered with a split-thickness skin graft harvested from the right thigh. However, during the hospitalization period, despite receiving appropriate psychiatric treatment, the patient experienced an episode of psychomotor agitation with self-mutilation tendencies. Consequently, he inflicted injuries to the cranial extremity, resulting in bleeding at the surgical site, subsequent ischemia, and eventual necrosis of the skin and fascial flap.

Microbiological analysis of the patient’s samples revealed a polymicrobial infection, specifically categorized as oculo-palpebral fasciitis type I. Acinetobacter cloacae with susceptibility to Tobramycin and Colistin were identified. The patient received intravenous Ceftriaxone and Colistin at a dosage of 3 million units every 24 h as part of the ongoing antibiotic therapy, according to the recommendations of the infectious disease specialist.

**Figure 4:**
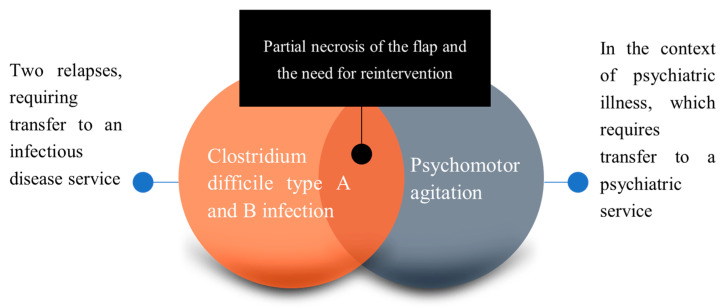
Postoperative evolution and the causes of flap necrosis.

Simultaneously, the patient developed a Clostridium difficile infection, characterized by the presence of both type A and B strains. This infection exhibited a recurrent course, necessitating the patient’s transfer to an Infectious Disease service for specialized management and treatment (Figure 4).

Following the patient’s psychiatric stabilization and the successful treatment of the *Clostridium difficile* infection, the patient returned to the Plastic Surgery clinic for the continuation and completion of surgical treatment.

**Figure 5:**
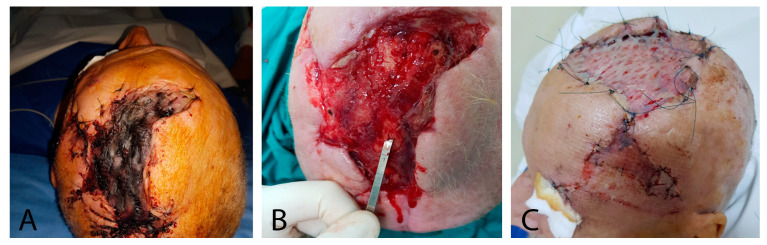
(**A**) Parieto-occipital fascial flap necrosis. (**B**) Surgical approach after parieto-occipital fascial flap necrosis—periosteal removal. (**C**) Immediate postoperative results.

Under general anesthesia with orotracheal intubation, the necrotic area (the temporal fascial flap) was excised, and the resulting defect, after bone deperiosting, was addressed through the application of a free split-thickness graft, harvested using an electrodermatome from the upper inner thigh of the right leg (Figure 5).

Following extensive medical care and close monitoring, the patient’s condition significantly improved, ultimately leading to their discharge after a total of 40 days of hospitalization.

A collaborative and multidisciplinary approach was adopted throughout the patient’s hospitalization, ensuring continuous communication and co-ordination among various departments, including Diabetes; Ear, Nose, and Throat (ENT); Ophthalmology; Infectious Diseases; and Psychiatry. This concerted effort allowed for comprehensive and integrated care, addressing the diverse medical needs and ensuring the optimal management of the patient’s condition.

**Figure 6:**
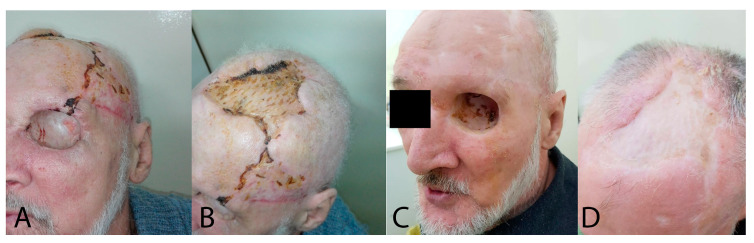
(**A**,**B**) Postoperative results at 6 months. (**C**,**D**) Postoperative results at 1 year.

Despite the ophthalmologist’s recommendation for the patient to return to the Ophthalmology Department to initiate the necessary procedures for an ocular prosthesis, the patient has not returned to the hospital (Figure 6). The last follow-up examination the patient attended was conducted two years after the surgical procedure.

### 2.2. Systematic Review

**Figure 7:**
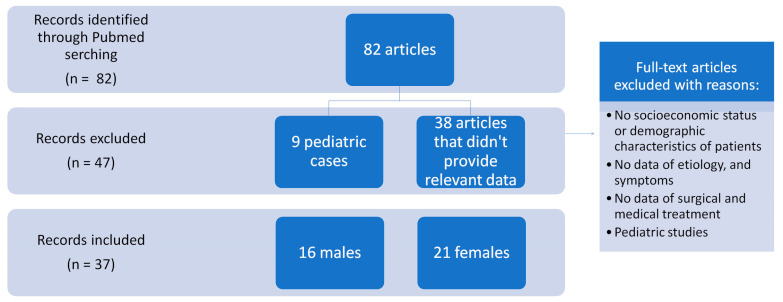
Search process and records included and excluded.

We conducted a comprehensive systematic review in adherence to the guidelines provided by “Preferred Reporting Items for Systematic Reviews and Meta-Analyses” (PRISMA) (Figure 7).

The primary objective of this review was to evaluate the existing literature on necrotizing fasciitis, particularly in the context of facial infections among adult populations. To ensure a thorough investigation, a meticulous search was performed in the PubMed database, utilizing the precise search terms “Necrotizing fasciitis”, “facial”, and “periorbital”. The search was specifically confined to case reports published from January 2018 to May 2023. The study was not registered.

Initially, abstracts of the identified articles were screened, and. subsequently, full-text articles were obtained, if available, for a more detailed assessment of their relevance by the authors. In order to maintain the focus of the review, several exclusion criteria were applied. Articles that primarily focused on pediatric cases, those that addressed different aspects other than necrotizing fasciitis, those that examined necrotizing fasciitis occurring in other anatomical regions, those lacking clear localization information, or those that failed to provide sufficient patient-related details were excluded from the final analysis.

A meticulous iterative process was employed to ensure accurate data extraction, and Microsoft Excel was utilized for basic statistical analysis of the extracted data. This methodical approach allowed for a comprehensive evaluation of the available literature and enabled the synthesis of meaningful findings.

## 3. Results

### Systematic Review

During the initial search process, 82 articles were identified as potentially relevant to the study. Among these articles, one was published in a language other than English and was, subsequently, excluded from further analysis due to language barriers. This resulted in a final pool of 81 articles that were deemed eligible for assessment.

Upon careful evaluation, it was determined that 9 articles focused on pediatric cases, rendering them unsuitable for the current investigation. Additionally, 28 articles explored different aspects which deviated from the specific objective of this study. Moreover, 10 articles failed to provide any relevant data, thereby disqualifying them from inclusion in the analysis.

Ultimately, a total of 34 articles met the established criteria and were included in the study. These articles included case reports of adult patients with facial necrotizing fasciitis. It is worth noting that most of the included studies presented a single case report, while a subset of studies featured two or more case reports, with a total of 38 cases, including our case (Table 1 and Table 2).

Demographic characteristics: A total of 38 cases were included in the analysis, consisting of 16 (42.1%) males and 21 (57.9%) females. The mean age of the participants was 49.71 years, with a standard deviation of 17.21, indicating a moderate degree of variability in the age distribution (Table 1).

Etiology of lesions: Among the observed cases, the predominant mechanism leading to the development of lesions was trauma, as reported in 12 instances (41%). This was followed by odontogenic causes in 8 cases (27%), dermatological conditions in 4 cases (14%), surgical procedures in 3 cases (10%), and other causes in 3 cases (10%). The etiology of lesions remained unidentified in 11 cases (38%).

Associated systemic disorders: Cases analyzed were assessed in search of patients comorbidites. Among the patients, 19 cases (50%) exhibited comorbidities, while 8 cases (21%) did not have any significant medical history, and the medical history of 5 cases (13%) could not be accurately determined. When considering the frequency of associated systemic disorders, the most prevalent conditions were diabetes mellitus, documented in 12 cases (32%); hypertension, observed in 10 cases (26%); chronic alcohol abuse, present in 8 cases (21%); and hepatic disease, identified in 4 cases (11%). Furthermore, cancer was reported in 3 cases (8%), kidney disease in 2 cases (5%), and autoimmune and genetic diseases in 3 cases (8%). Therefore, immunosuppression was noted in 16 cases (42%).

Localization: Facial necrotizing fasciitis was most commonly found in the periorbital area, followed by cervical region, eyelids, submandibular area, temporal area, cheeks, submental area, malar and mandibular areas, lips, forehead, glabellar area, and chin and thoracic region.

Initial clinical manifestations: The most common signs and symptoms were swelling and pain, with all patients reporting them as their initial clinical manifestations, respectively. Fever, edema, erythema, and purulent discharge were also reported in most cases and dysphagia in some cases.

Diagnosis: A Laboratory Risk Indicator for Necrotizing Fasciitis score was assessed in most cases with values between 6 and 10. Bacterial culture was performed in all cases. A meticulous assessment was performed to elucidate the microbial patterns and identify the associated micro-organisms. Out of the total cases, a monomicrobial pattern was observed in 22 cases (58%), while a polymicrobial pattern was identified in 11 cases (29%). The microbial pattern was not specified in 3 cases (8%), and, in 2 cases (5%), cultures yielded negative results. The most common micro-organisms isolated in order of frequency were: Group A *beta-haemolytic streptococci* (12 cases), *MRSA* (Methicillin-resistant Staphylococcus aureus) (7 cases), *Klebsiella pneumoniae* (7 cases), *Pseudomonas aeruginosa* (3 cases), *Staphylococcus epidermidis* (3 cases), *Streptococcus* milleri group (3 cases), *Acinetobacter* sp. (2 cases), *Enterobacter cloacae* (2 cases), and fungi (2 cases). Other bacteria like *E. faecalis*, *Eikenella corrodens*, *Klebsiella oxytoca*, *Prevotella nigrescens*, *Staphylococcus lugdunensis*, *Stenotrophomona smaltophilia*, and *Veillonella parvula* were also identified in singular cases.

Surgical treatment: Surgical debridement was required in all cases. In most cases, the defect was covered by skin graft (13 cases) and flaps (8 cases). In 3 cases, negative-pressure wound therapy (NWPT) was used.

## 4. Discussion

Necrotizing fasciitis is a severe condition with a rapid course and high mortality risk (35%) [14,15,16,17]. Periocular involvement is rarely reported in the literature, being both uncommon and debilitating, sometimes resulting in the need for exenteration of the affected eye globe [4,11,18,19]. Despite the relatively lower mortality rate in these cases (ranging from 6 to 14%), the context of this pathology can be posttraumatic or postoperative. In the reported case, the patient, homeless, fell victim to an assault [20,21]. The onset of the disease is favored by the presence of comorbidities such as diabetes mellitus, malignant tumors, and immunosuppression, as reported in the literature [22,23]. Similarly, in the presented case, periocular necrotizing fasciitis was observed in a patient with poor living conditions (homeless), and multiple comorbidities, including diagnosed diabetes mellitus, cardiovascular disease, and psychiatric pathology [22,24].

Microbial agents present in such pathologies are diverse, including Group A beta-hemolytic streptococci, MRSA (Methicillin-resistant *Staphylococcus aureus*), *Klebsiella pneumoniae*, *Pseudomonas aeruginosa*, *Staphylococcus epidermidis*, *Streptococcus milleri group*, *Acinetobacter* sp., *Enterobacter cloacae*, and fungi. In the reported case, Acinetobacter cloacae was implicated, and, during hospitalization under antibiotic treatment, the patient developed a secondary infection with *Clostridium difficile*, leading to a transfer to an infectious disease unit [12,25,26,27,28,29,30,31]. This led to the discontinuation of surgical treatment, exacerbating the immunosuppressive state and pre-existing imbalances [24,32,33,34,35].

The patient’s psychiatric condition further complicated the surgical treatment progression, as there was an episode of psychiatric distress with self-harm, leading to fascial tissue necrosis, consequently necessitating a subsequent surgical intervention to cover the exposed bone.

In addition to a clinical examination with the detection of characteristic signs and symptoms, including the presence of cutaneous emphysema, the diagnosis of periocular necrotizing fasciitis requires imaging examination, such as an MRI, to confirm or support the diagnosis [2,3,36,37]. Surgical treatment is always necessary and must be combined with antibiotic treatment tailored to the sensitivity of the germs involved in the etiology of the disease. Surgical intervention must often be aggressive, with extensive devitalized tissue excision, often resulting in large soft tissue defects [4,6]. In the reported case, due to the high risks of cerebral involvement, exenteration of the eye globe was necessary, an incapacitating operation for the patient [38].

In all cases from the literature, the covering of the remaining soft tissue defects post-necrectomy required various reconstructive techniques, from split-thickness skin grafts to local or distant flaps (Table 2). These techniques are always selected based on the size and location of the defect, with respect, if possible, to the aesthetic units of the face, aiming for the best possible functional and aesthetic outcome.

Given the severity of the disease, the localization, and the involvement of various anatomical regions of the face, a multidisciplinary surgical approach (involving plastic surgeons, ophthalmologists, and craniofacial surgeons) becomes necessary. In the described case, the complexity of the disease, the complications, and the patient’s comorbidities required collaboration with specialists in infectious diseases and psychiatry.

Despite the reduced mortality in cases of periocular necrotizing fasciitis, the remaining sequelae of such a disease can often be significant, both aesthetically and functionally, sometimes leading to disabilities, vision impairment, or even loss of the eyes.

## 5. Conclusions

Necrotizing fasciitis, a severe condition with a high mortality risk, necessitates thorough comprehension, particularly concerning the possibility of uncommon localizations, such as facial involvement, notably periocular. Additionally, obtaining a precise and comprehensive patient history enables the identification of predisposing factors for this pathology.

Early diagnosis (both clinical and imaging) can lead to prompt intervention, minimizing invasiveness and complexity, resulting in reduced patient hospitalization time and fewer functional and aesthetic sequelaes. Successful outcomes in these instances rely heavily on interdisciplinary collaboration, ensuring the patient receives a comprehensive and integrated treatment.

## Figures and Tables

**Table 1:** Parameters of the cases included in the facial necrotizing fasciitis systematic review.

	Year	Reference	Gender and Age	Etiology	Alcohol Use	Associated Pathologies	Symptoms
1	2023	Sarah Nyirjesy et al. [7]	58, F	Decayed teeth	Unknown	Unknown	Right facial and neck swelling
2	2023	Nripen Gaur et al. [8]	30, M	Unknown	No	None	Gradual swelling of the right eyelids and adjacent skin
3	2022	Mosenia A et al. [9]	39, M	Trauma, decayed tooth	Yes	Heavy alcohol use	Left-sided periorbital swelling pain, erythema
4	2022	Da-Woon Lee et al. [10]	43, M	Hematogenic spread from lung and renal abscesses	No	Diabetes mellitus with insulin	Left hemifacial swelling involving the buccal and submandibular areas, fever
5	2022	Silverman et al. [11]	21, M	Trauma	Unknown	Unknown	Rigors and right-eye pain and swelling
6	2021	Ling Jin et al. [12]	48, M	Peritonsillar abscess	No	Diabetes mellitus with unstable control with insulin	Four-day sore in left throat and half-day dysphagia, and fever, cold, and fatigue without toothache
7	2021	Akshay J Reddy et al. [13]	44, M	Unknown	Yes	Heavy alcohol use	Swelling, tenderness, and erythema around his right eye, including the right side of his face
8	2021	Bülent Yazıcı et al. [14]	70, M	Trauma	No	Diabetes mellitus	Afebrile, fatigued, and in distress
9	2021	Amanda Cecchini et al. [15]	28, F	Dental abscess	No	No	Facial pain and swelling, headache, and vomiting
10	2021	Alice Rigby et al. [16]	65, F	Surgical carcinoma excision with reconstruction with fibula free flap	No	Type 2 diabetes, coronary artery disease, hyper- tension, hypercholesterolaemia and acid reflux, moderate differentiated squamous cell carcinoma (SCC),	Necrotic skin edges, necrotic muscles including the platysma, strap muscles, sternocleidomastoid, and anterior belly of digastric
11	2021	Yu-Kuei et al. [17]	62, M	unknown	Yes	hepatitis C and chronic kidney disease after kidney transplantation Under steroids and immunosuppressants, bilateral vitreous hemorrhage and tractional retinal detachment	Periorbital pain and erythematous eyelid swelling
12	2020	Pang-Yun Chou et al. [18]	44, M	Decayed tooth (molar self-extracted at home)	No	Diabetes mellitus, poor oral hygiene	Progressive left facial necrosis and blurred vision of the left eye, left periorbital pain, swelling, fever, and general malaise
13	2020	Hyeong Seop Kim et al. [19]	60, F	Bullous lesion in the upper lip	Unknown	Diabetes mellitus and chronic renal failure	Severe pain in the upper lip, cellulitis, upper lip gangrene, and necrosis
14	2020	Aman Negi et al. [4]	32, M	Trauma to the left side of face	Unknown	Unknown	Swelling, erythema, purulent discharge, fever, and blackish necrotic skin
15	2020	Grace Anne McCabe et al. [20]	56, F	Unknown	Yes	Hypertension, alcohol excess, and obesity	Periorbital swelling
16	2020	Rebecca A Compton et al. [21]	44, F	Four days after bathing in a public hot tub	Unknown	None	Periorbital swelling and pain
17	2020	Chloe Ft Ting et al. [22]	35, F	Trauma, subgaleal hematoma	Yes	Chronic alcohol abuse	Bilateral periorbital pain and swelling, hypotension, tachycardia
18	2020	Amir Ali Badri et al. [23]	19, M	Trauma, surgery	No	No	Swelling extending from submandibular to suprasternal area, with tender and erythematous skin
19	2020	Landeen Kelly et al. [24]	58, F	Unknown, immunosuppressed	No	Rheumatoid arthritis (tocilizumab), depression	Erythema, swelling, necrotic eyelids
20	2019	Karan NB et al. [25]	81, F	Stomatitis	No	right-side facial palsy, diabetes mellitus	Right eyelid edema and diplopia, swelling in the right maxillary area for 7 days in duration
21	2019	Haen P et al. [26]	57, F	Rhytidectomy	No	None	Inflammation and swelling of the face, with blisters and necrotic lesions
22	2019	Jinhwan Park et al. [27]	53, F	Unknown	Yes	Diabetus mellitus, chronic hepatitis B, liver cirrhosis, hepatocellular carcinoma	Swelling, erythema
23	2019	Nadal J et al. [28]	32, F	Unknown	Yes	Breast adenocarcinoma, chronic alcoholism	Right upper eyelid edema, redness and, severe pain
24	2019	Rouli Sud et al. [29]	52, F	Unknown	Unknown	Unknown	Bilateral painful lid swelling
25	2019	David Steybe et al. [30]	77, F	Bisphosphonate-related osteonecrosis, tooth extraction		Postmenopausal osteoporosis and type 2 diabetes	Severe, painful swelling affecting the right submandibular and submental region
26	2019	Jongweon Shin et al. [31]	79, F	Trauma	Unknown	Hypertension	Right-sided facial swelling
27	2018	Jaffer ZN et al. [32]	51, F	Unknown	No	Systemic lupus erythematosus (SLE) and Lynch syndrome	Bilateral periorbital swelling
28	2018	Sondo KA et al., review of 4 cases [33]	1. 43, M 2. 20, F 3. 53, F 4. 44, F	1. Trauma to zygomatic region 2. Infected boil 3. Micropapule itching of the left cheek 4. A furuncle on the left cheek	1. No 2. No 3. No 4. No	1. Diabetes mellitus 2. Drepanocytosis 3. None 4. None	1. Facial paralysis, lymphadenopaty 2. Pain and edema 3. Edema, facial asymmetry, ulceration of the left cheek covered with a purulent coating and necrotic crusts 4. edema, pain, fever
29	2018	Tri Rejeki Herdiana et al. [34]	69, F	Left preseptal eyelid infection post surgery	No	Aesthetic upper and lower blepharoplasty on both sides	fever and left eyelid swelling, partial gray-tan discoloration of the involved eyelid of over 3 days’ duration
30	2018	Bekir Selim Bağli et al. [35]	60, M	Odontogenic infection	No	Unknown	Facial pain and swelling
31	2018	Paul Andrei Ţenţ et al. [36]	47, F	Trauma	Yes	Heavy alcohol and tobacco use; chronic psoriasis vulgaris	Facial pain and swelling
32	2018	Gillespie et al. [37]	44, M	ACE inhibitor-induced facial angioedema	Yes	Type 2 diabetes mellitus, primary hypertension, and a history of substance abuse	Tender and swollen eyelid nodule consistent with a hordeolum
33	2018	Deneubourg D et al. [38]	30, F	Trauma	No	no	Bilateral painful palpebral swelling; palpebral necrosis of the superior and inferior left eyelids, chemosis, redness and facial edema extending to the upper chest and the contralateral eyelids; fever
34	2018	CongRan et al. [39]	24, M	Removal of an ingrown hair from his chin	No	Emtricitabine/tenofovir for HIV pre-exposure prophylaxis	Fever, as well as facial pain, erythema, and swelling

M = male; and F = female.

**Table 2:** Antibiotic therapy and surgical treatment of the cases included in the facial necrotizing fasciitis systematic review.

Reference	Empiric Antibiotic Therapy	Isolated Micro-Organism	Definitive Antibiotic Therapy	Multidisciplinary Team	Surgical Treatment
Sarah Nyirjesy et al. [7]	Vancomycin, ampicillin-sulbactam, and fluconazole for a planned duration of 4 weeks	Unknown	Vancomycin, ampicillin-sulbactam, and fluconazole	Otolaryngology, Ophtalomology	Surgical debridement, negative-pressure therapy, 3D-printed, patient-specific wound splint, pectoralis muscle flap, split-thickness skin graft, and right paramedian forehead flap
Nripen Gaur et al. [8]	Unknown	Gram-positive cocci	Ceftriaxone 1 g 12 hourly and amikacin 400 mg 12 hourly	Unknown	Surgical debridement
Mosenia A et al. [9]	Vancomycin and (piperacillin-tazobactam), Clindamycin.	*Staphylococcus epidermidis* *Streptococcus milleri group* *Staphylococcus lugdunensis.*	Ceftriaxone and metronidazole for 7 days	Infectious Disease, Plastic and Reconstructive Surgery, Oral and Maxillofacial Surgery	Surgical debridement, maxillary antrostomy, ethmoidectomy, and extraction of the infected tooth. Repair with a split-thickness skin graft.
Da-Woon Lee et al. [10]	Meropenem 1 g every 8 h and levofloxacin 750 mg per day	*K. pneumoniae*	Cefotaxime + Ceftriaxona 2 g/d	Infectious Disease, Plastic and Reconstructive Surgery	Surgical debridement and decompression
Silverman et al. [11]	Unknown	Unknown	Unknown	Unknown	Surgical debridement
Ling Jin et al. [12]	Vancomycin, piperacillin sulbactam, and ornidazole	*Klebsiella oxytoca* *Streptococcus constellatus* *Candida albicans and candida guilliermondii)*	Meropenem Tigecycline Fluconazole	Infectious Disease, Plastic and Reconstructive Surgery	Negative-pressure drain tube, surgical debridement
Akshay J Reddy et al. [13]	Unknown	*Streptococcus pyogenes*	Unknown	Infectious Disease, Plastic and Reconstructive Surgery, Maxillofacial Surgery, ICU	Surgical debridement and wound negative-pressure therapy
Bülent Yazıcı et al. [14]	Ampicillin-sulbactam (6 g/day, intravenous) Ciprofloxacin (1200 mg/day, intravenous	*Staphylococcus aureus,* *Streptococcus* *p* *arasanguinis,* *Enterobacter cloacae*	Unknown	Ophtalmology, Plastic and Reconstructive Surgery	Surgical debridement. Upper and lower eyelid reconstructions with skin grafts.
Amanda Cecchini et al. [15]	900 mg intravenous (IV) clindamycin, IV vancomycin	*Streptococcus anginosus, group F streptococcus,* *Eikenella corrodens, Prevotella, and Bacteroides species*	A two-week course of linezolid and clindamycin	Oral Surgery and Otolaryngology	Surgical debridement
Alice Rigby et al. [16]	Teicoplanin, clindamycin, aztreonam, and gentamicin	*G* *ram-negative anaerobes, including veillonella parvula* *, pseudomonasaeruginosa*	Teicoplanin, clindamycin, ciprofloxacin	Department of Oncology	Surgical debridement, pedicle flap (pectoralis major)
Yu-Kuei et al. [17]	400 mg intravenous teicoplanin every other day and 2000 mg ceftriaxone once a day	*Pseudomonas aeruginosa*	2000 mg ceftazidimeonce a day	Ophtalmology	Surgical debridement and anterior orbitotomy
Pang-Yun Chou et al. [18]	Oxacilin Ceftazidim	*Pseudomonas aeruginosa and S. aureus* *Klebsiella pneumoniae*	Vancomycin imipenem/cilastatin.	Maxillofacial Surgery, Plastic and Reconstructive Surgery	Surgical debridement. Left medial thigh meshed split-thickness skin graft.
Hyeong Seop Kim et al. [19]	Unknown	*Klebsiella pneumoniae* *Methicillin-resistant Staphylococcus aureus (MRSA)*	Ceftriaxone and metronidazole	Department of Dermatology	Surgical debdridement, Abbe flap
Aman Negi et al. [4]	Unknown	Group A *beta-haemolytic streptococci*	Intravenous penicillin G and clindamycin	Department of General Surgery	Surgical debridement and intermediate-thickness skin grafting
Grace Anne McCabe et al. [20]	Unknown	Group A *Streptococcus* (GAS).	Vancomycin, benzylpenicillin, meropenemandclindamycin	Ophthalmology and Plastic Surgery	Surgical debridement, full-thickness skin graft
Rebecca A Compton et al. [21]	Unknown	Group A *Streptococcus*	Unknown	Ophtalmology	Surgical debridement. Reconstructed with full-thickness grafts
Chloe Ft Ting et al. [22]	IV flucloxacillin, clindamycin	*S. pyogenes*, methicillin-resistant S. aureus (MRSA)	Penicillin and clindamycin, cephalexin	Ophthalmology, Infectious Diseases Services	Surgical debridement, full-thickness skin graft
Amir Ali Badri et al. [23]	Unknown	*Staphylococcusaureus*	Vancomycin and meropenem	Department of Oral and Maxillofacial Surgery	Surgical debridement, deltopectoral flap
Landeen Kelly et al. [24]	Unknown	*Streptococcus pyogenes,* MRSA	Penicillin, vancomycin	Ophtalmology, Otolaryngology	Surgical debridement
Karan NB et al. [25]	Ciprofloxacin Trimethoprim-sulfamethoxazole	*S. maltophilia*	Unknown	Maxillofacial Surgery, Plastic and Reconstructive Surgery, Infectious Disease	Surgical debridement, enucleation of the right eye. Microsurgical rectus abdominis myocutaneous (TRAM) flap reconstruction.
Haen P et al. [26]	Unknown	Group A streptococcus	Unknown	Oral and Maxillofacial Surgery	Surgical debridement, bilateral orbitotomy, functional endoscopic sinus surgery
Jinhwan Park et al. [27]	Tazime (Ceftazidime, 1 g/8 h), vancomycin 1 g/24 h, Clindamycin 600 mg/8 h	*K. pneumoniae*	Ceftazidime	Opthalmology	Surgical debridement, endoscopic sinus surgery, transverse abdominis myocutaneusflap
Nadal J et al. [28]	Tazocillin 1 g × 3/day, Vancomycine 30 mg/kg/day and Clindamycine 600 mg × 3/day intravenously	Group A strep- tococcus	Unknown	Opthalmology	Surgical debridement
Rouli Sud et al. [29]	Amikacin	Gram-negative bacilli, *Kleibsella pneumoniae*	Amikacin	Ophtalmology	Surgical debridement
David Steybe et al. [30]	Penicillin (10 Mio. IU once a day) and metronidazole (500 mg twice a day)	*Prevotella nigrescens* resistant to penicillin but sensitive to metronidazole	Cefuroxime (1.5 g twice a day) andmetronidazole (500 mg twice a day)	Oral and MaxillofacialSurgery	Surgical debridement, surgical resection of the necrotic bone, reconstruction plate was inserted for stabilization of the mandible
Jongweon Shin et al. [31]	Ceftazidime and clindamycin	Group A *beta-hemolytic Streptoccus*	Ceftriaxone and clindamycin	Infectious Disease Department	Surgical debridement
Jaffer ZN et al. [32]	piperacillin–tazobactam 4.5 g intravenously every 8 h and vancomycin 1500 mg intravenously every 12 h, clindamycin 900 mg	Group A Streptococcus (*Streptococcus pyogenes*)	5 days of intravenous piperacillin–tazobactam	Ophtalmology, Plastic and Reconstructive Surgery, Intensive Care Unit	Surgical debridement healing; granulation tissue with oculoplastic follow-up
Sondo KA et al., review of 4 cases [33]	1. Ceftriaxone 2 g/day 2. Amoxicilline-acide clavulanique 1 g/8 h and gentamicine 160 mg/day 3. Oxaciline 4. Unknown	1. Non-groupable Streptococcus 2. *Staphylococcus aureus* 3. *S. epidermidis* 4. Blood cultures negative	1. Ceftriaxone 2 g/day, gentamicine 160 mg/day 2. Amoxicilline 6 g/day and ciprofloxacine 1 g/day 3. Amoxicilline—acide clavulanique 4. Amoxicilline—acide clavulanique 1g/8h	Infectious Disease	Surgical debridement
Tri Rejeki Herdiana et al. [34]	Flomoxefsodium, 1.8 g/day of intravenous clindamycin, 6 g/day of ampicillin, and 1.2 g/day of vancomycin	*Streptococcus pyogenes*	1.8 g/day of intravenous clindamycin, 6 g/day of ampicillin, oral levofloxacin	Oculoplastic specialist, intensivist, infection disease specialist, endocrinologist, and dermatologist	Surgical debridement, skin graft, and local skin flaps
Bekir Selim Bağli et al. [35]	Unknown	Unknown	Unknown	Department of Underwater and Hyperbaric Medicine, Department of Otorhinolaryngology	Surgical debridement and hyperbaric oxygen (HBO2) therapy
Paul Andrei Ţenţ et al. [36]	Colistin 3 million IU/8 h Vancomycin 1000 mg, Imipenem 500 mg, and Metronidazole 500 mg	*Staphylococcus epidermidis* *Klebsiella sp.* *Acinetobacter sp.*	Colistin administered as an intravenous injection of 3 million IU every 8 h in association with the previous antibiotics	Oral and Maxillofacial Surgery; Plastic Surgery Department	Surgical debridement, flaps (pedicled trapezius musculocutaneous and rotated scalp flaps), and two free skin grafts
Gillespie et al. [37]	Vancomycin, piperacillin/tazobactam, clindamycin, and steroid, clindamycin, meropenem, and micafungin	*S. pyogenes*	Vancomycin, piperacillin/tazobactam, clindamycin, and steroid	Plastic Surgery Department, Ophtalmology	Surgical debridement, NPWT for 2 weeks, bilateral upper lid full-thickness skin grafting. NPWT was continued for 6 days after skin graft placement with dressing changes every 2 days.
Deneubourg D et al. [38]	1 g of intravenous amoxicillin and clavulanic acid, piperacillin-tazobactam, gentamicin and clindamycin	Group A *beta- haemolytic streptococcus (Stresptococcus pyogenes)*	Oral clindamycin (1800 mg in 3 takes) and amoxicillin 9 g per day from day 4 today 10, 12 g a day from day 11 today 25) and followed by oral amoxicillin (6 g per day from day 26 to 41)	Stomatology Maxillofacial and Plastic Surgery, Infectious Disease	Surgical debridement, full-thickness skin graft
CongRan et al. [39]	Unknown	Clindamycin-resistant and methicillin-resistant strain of *Staphylococcus aureus*	Unknown	Otolaryngology	Surgical debridement

NPWT = negative-pressure wound therapy, ICU = Intensive Care Unit, IU = international unit, and g = grams.
